# Supplementary material for: Comparative Rumen Metagenome and CAZyme Profiles in Cattle and Buffaloes: Implications for Methane Yield and Rumen Fermentation on a Common Diet
Source: Microorganisms. 2023 Dec 27;12(1):47. doi: 10.3390/microorganisms12010047 (PMC10818812; doi:10.3390/microorganisms12010047)
Supplement: Supplementary file 1 [file microorganisms-12-00047-s001.zip › microorganisms-2737754-SM.pdf]

Supplementary

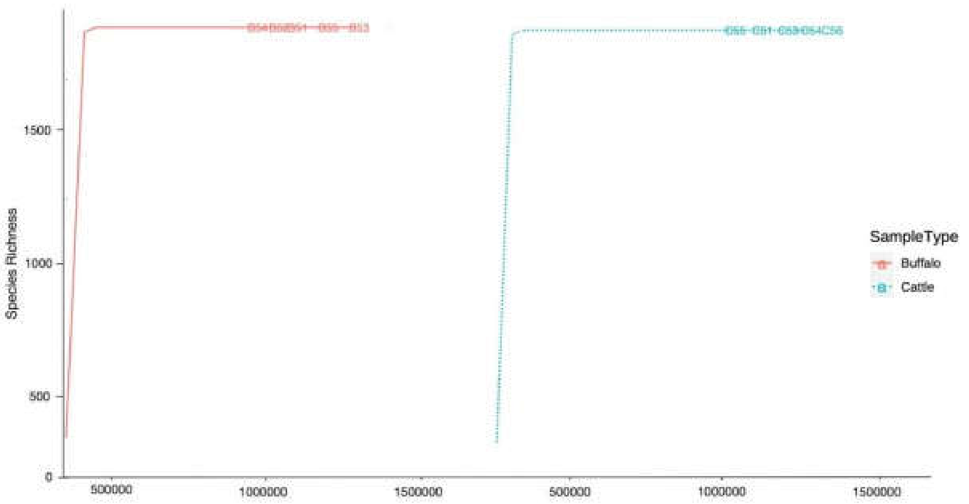

Figure S1. Rarefaction curves of cattle and buffaloes rumen metagenome.

Supplementary File S1: Read stats and taxonomic assignments of the rumen microbiota

Supplementary File S2: CAZymes abundances
